# Label-free and real-time imaging of dehydration-induced DNA conformational changes in cellular nucleus using second harmonic microscopy

**DOI:** 10.1038/srep07416

**Published:** 2014-12-10

**Authors:** Shuangmu Zhuo, Ming Ni

**Affiliations:** 1 Fujian Provincial Key Laboratory for Photonics Technology, Key Laboratory of OptoElectronic Science and Technology for Medicine of Ministry of Education, Fujian Normal University, Fuzhou 350007, China; 2 Institute of Bioengineering and Nanotechnology, 31 Biopolis Way, The Nanos, Singapore 138669, Singapore

## Abstract

Dehydration-induced DNA conformational changes have been probed for the first time with the use of second harmonic microscopy. Unlike conventional approaches, second harmonic microscopy provides a label-free and real-time approach to detect DNA conformational changes. Upon dehydration, cellular DNA undergoes a transition from B- to A-form, whereas cellular nuclei change from invisible to visible under second harmonic microscopy. These results showed that DNA is a second order nonlinear optical material. We further confirmed this by characterizing the nonlinear optical properties of extracted DNA from human cells. Our findings open a new path for SHG imaging. DNA can change its conformations under many circumstances. For example: normal cells turning into cancerous cells and drug molecules binding with DNA. Therefore, the detection of DNA conformational changes with second harmonic microscopy will be a useful tool in cancer therapy and new drug discovery.

Imaging of cellular nucleus can be easily achieved by 4′, 6-diamidino-2-phenylindole (DAPI) and Hoechst dyes. However, these fluorescence dyes cannot be used to monitor DNA conformational changes inside cell nucleus. Understanding such changes is crucial for disease diagnosis, cell state monitoring, and tracing genetic modification in cancer cells. So far, medical diagnostic tools, such as Fourier transform infrared (FTIR) spectroscopy and surface enhanced Raman spectroscopy (SERS) have been applied successfully for these purposes^1^,^2^. Although existing techniques and fluorescent probes are quite powerful, nobody use DNA itself for as a probe to visualize cell nucleus and for other bioimaging applications. Of particular interest, DNA will undergo conformational change from the native B-form to A-form when cells are dehydrated^1^. A-form DNA, a more stabilized conformation, has been hypothesized as a cellular defense mechanism^3^,^4^. It has been reported that the B to A transition plays an important role in response to UV radiation^5^,^6^,^7^, chemical toxicity^8^, and other forms of DNA damage^3^. In general, monitoring the cellular DNA conformational change from B to A transition is crucial in cell biochemistry and understanding the evolutionary pathway of nucleic acids.

Here we report, for the first time, the detection of dehydration-driven DNA conformational changes inside eukaryotic cells using second harmonic generation (SHG) microscopy.

## Results and Discussion

**Figure 1:**
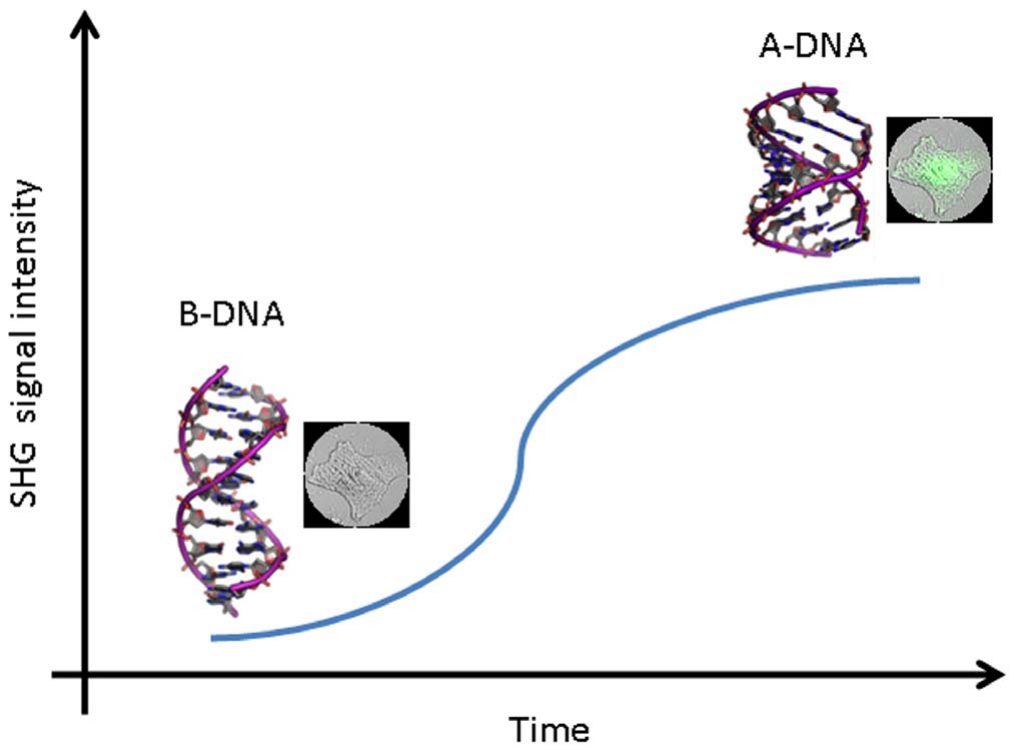
SHG intensity of cellular DNA varies during the dehydration of HeLa cells. The conformation of cellular DNA undergoes B to A transition; A-form DNA is demonstrated to be SHG-active whereas B-form DNA is not. The excitation wavelength *λ_ex_* was 850 nm and the excitation power was 4 mW.

For this label-free and real-time detection approach, we don't use any exogenous probes or fluorescent dyes but fully depend on the SHG signals generated from cellular DNA. During dehydration, we were able to detect DNA inside cell nucleus changing status from SHG-inactive to SHG-active, as shown in Figure 1. This observed phenomenon is attributed to the DNA conformational change.

In this work, A-form DNA is demonstrated to be SHG-active whereas B-form DNA is not. We chose dehydration-driven DNA conformational change as a model because similar trend was reported by means of FTIR. However, A-form and B-form DNA can only be discerned by FTIR when principal component analysis is in use. This is because the shifts of FTIR bands were small.

**Figure 2:**
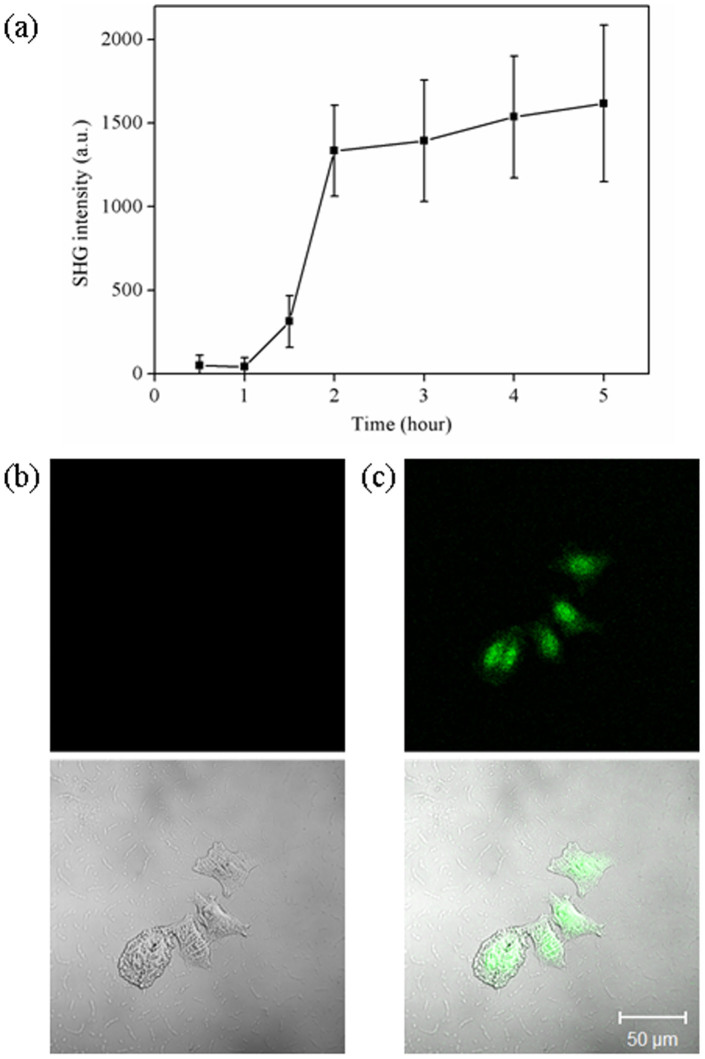
SHG intensity from HeLa cells varied with increasing dehydration time. (a) time course of SHG intensity; (b) SHG and phase contrast images of HeLa cells at 0.5 hour; (c) SHG and phase contrast images of HeLa cells at 4 hour. The excitation wavelength *λ_ex_* was 850 nm and the excitation power was 4 mW.

On the contrary, when we applied SHG, we can see black-and-white difference for the same change. This clearly shows the advantages for using SHG over FTIR. The time course of the SHG signals generated from the DNA inside HeLa cell nucleus is shown in Figure 2. A sigmoidal shape was observed which is corresponding to the B to A transition of double-stranded DNA. Similar trend was observed in the literature when peak position of the anti-symmetric phosphate stretching vibration from 1224 to 1236 cm^−1^ was shifted with decreasing/increasing hydration^1^. At 1.5 hour, cell nucleus suddenly became visible under second harmonic microscopy (Figure 2). It is worth noting that no DAPI or other dyes were added. All these results demonstrated that the dehydrated DNA is a SHG-active material. Under natural hydrated condition, it is SHG-inactive; whereas under dehydration, it is undergoing structural rearrangement and become SHG-active.

Besides FITR, SERS is another powerful spectroscopic technique that has been used to detect the DNA conformational change^2^. Panikkanvalappil et al. has successfully used it to differentiate cancerous and healthy cells^2^. They observed the Raman marker bands red-shifted in cancerous cells after dehydration whereas no such shift was observed in healthy cells. SERS can provide structural information of DNA and almost every molecule. It is a noninvasive and sensitive tool, offering several important advantages over other medical diagnostic tools. However, in this case, DNA had to be extracted from cells and silver nanoparticles were added to DNA to enhance the SERS signals. Moreover, certain knowledge is required to interpret the Raman spectra. SHG is much more straightforward compared to SERS: 1) DNA was detected in the intact cells; 2) no silver nanoparticles or other reagents are needed; 3) no expertise on Raman or IR spectra interpretation is needed.

SHG, also known as frequency doubling, is a nonlinear optical process in which photons (ω_1_) interact with the nonlinear optical media and are converted into new photons with twice the energy (2ω_1_)^9^. Unlike other nonlinear optical process, only noncentrosymmetric structures are able to emit SHG signals^9^. Such examples in cells, tissues, and organisms, include microtubules, collagen, and muscle myosin. In particular, collagen is SHG-active and people have found that the molecular structures of collagen in healthy and diseased tissue are different. These enable SHG as a label-free technique to detect skin diseases, liver fibrosis and delineate tumor^10^,^11^,^12^,^13^. Our results indicated that DNA is also SHG active under certain circumstances (e.g. dehydration). This can enhance the capability of SHG as a label-free imaging tool.

**Figure 3:**
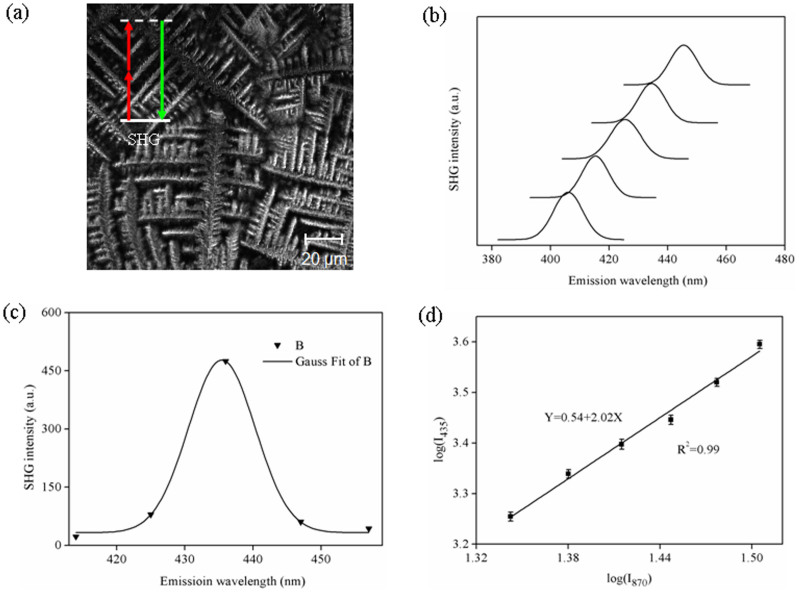
Characterization of DNA as a second order nonlinear optical material. (a) SHG images of extracted DNA; (b) SHG excitation wavelength tunability, from top to bottom, λ_ex_ = 810, 830, 850, 870 and 890 nm; (c) The fitting of emission wavelength versus SHG intensity (λ_ex_ = 870 nm). It fits well with a Gauss distribution with a narrow band width (full width at half maximum, FWHM) around 10 nm; (d) The log-log plot of the intensity of excitation and emission. The slope is 2, which corresponds to a second order. The excitation power was <5 mW.

Cell nucleus contains not just DNA, but also histone proteins and polysaccharides. To rule out SHG signals come from other components of cell nucleus, DNA was extracted from human cells and tested whether DNA is a second order nonlinear optical material. Extracted DNA was drop-coated on a Glass Bottom Dish and examined by second harmonic microscopy. Pine-needle shaped DNA molecules were clearly seen under SHG microscopy (Figure 3a). Distinct SHG emission peaks were observed at exactly one half of the excitation wavelengths (Figure 3b). The full width at half-maximum of Gaussian distribution obeys a 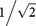 relationship to the corresponding wavelength profile of the fundamental beam (Figure 3c). The dependence of signal intensity versus excitation intensity is consistent with nonlinear 2^nd^ order optical up-conversion (Figure 3d). Based on these, DNA is indeed a second order nonlinear optical material.

Since SHG is a coherent process, we can continuously monitor HeLa cells for hours. The SHG activity of DNA does vary significantly. This is the other advantage of SHG compared to conventional fluorescence microscopy. It simply does not photo-bleach.

By virtue of its nonlinear optical properties, DNA can be used as a second harmonic probe. DNA is also an interesting building block for tissue engineering scaffold. DNA hydrogels and many DNA-based biomaterials have been developed in recent years^14^,^15^. DNA can either be extracted from biological samples or synthesized in the laboratory. It also contains the genetic codes with many useful biological functions. In this work, we investigated the nonlinear optical properties of DNA, which sheds new lights on this material.

In conclusion, we used a cell dehydration model to study the DNA conformational change by second harmonic microscopy in manner of label-free and real-time. DNA is shown to be SHG-active under the dehydration stage. This opens a new path for label-free SHG imaging. DNA carries the most important genetic information for every species on earth. Therefore, it widens the applications of DNA in SHG imaging. With the advance of instrumentation, second harmonic microscopy will become more sensitive and intricate difference in DNA molecular structure between diseased and healthy stages will be able to be discerned. Furthermore, SHG is a coherent process and caused no photo-bleaching or photo-damaging. Finally, it should be pointed out that although other processes such as multiphoton absorption will cause sample damage when the SHG excitation light is shined on the sample, the use of laser at a relatively low power level has simply made a longer observation period^16^,^17^.

## Methods

SHG imaging was achieved by the previously reported nonlinear optical system^13^. Briefly, a mode-locked femtosecond Ti: sapphire laser (110 fs, 76 MHz), tunable from 700 nm to 980 nm (Coherent Mira 900-F) was coupled to a commercial laser scanning microscopic imaging system (Zeiss LSM 510 META, Jena, Germany). The polarization direction of the laser light is the horizontal polarization. An oil immersion objective (×40 and NA = 1.3) was used focus the excitation beam into the sample and collect the backscattered SHG signals. The average laser power at the sample was controlled at <5 mW and no photobleaching was observed at this low power level^16^,^17^.

The samples used in this study were HeLa cells and DNA extracted from HeLa cells. Specifically, HeLa cells were seeded in a chamber slide (Nunc) and fed with growth media. The next day, the media were completely removed and the chamber slide was immediately placed on the microscope stage for taking SHG images. Moreover, DNA was extracted from HeLa cells using the DNAzol reagent (Life Technologies, Gaithersburg, MD, USA) as per the manufacturer's instruction. Briefly, HeLa cells grown in monolayer were lysed by adding DNAzol directly to a culture plate. Following centrifugation of the lysate, DNA was precipitated from the supernatant with ethanol and recovered by spooling. The DNA pellet was washed with 75% ethanol and solubilized in 8 mM NaOH. Then, for SHG imaging, DNA was placed in the Glass Bottom Dish (MatTek, coverglass: 0.085–0.13 mm) and allowed to air-dry at ambient condition. It should be noted that compared to the native DNA in cells, the conformational change may occur in the extracted DNA during air-drying.

## Author Contributions

S.Z. and M.N. designed research, performed research, analyzed data and wrote the paper.
